# Supplementary material for: Long-Term Hepatitis B Virus Infection Induces Cytopathic Effects in Primary Human Hepatocytes, and Can Be Partially Reversed by Antiviral Therapy
Source: Microbiol Spectr. 2022 Feb 16;10(1):e01328-21. doi: 10.1128/spectrum.01328-21 (PMC8849052; doi:10.1128/spectrum.01328-21)
Supplement: SUPPLEMENTAL FILE 6 — Supplemental material. Download SPECTRUM01328-21_Supp_6_seq2.pdf, PDF file, 3.5 MB [file spectrum01328-21_supp_6_seq2.pdf]

Supplementary Materials

Long-Term Hepatitis B Virus Infection Induces Cytopathic Effects in Primary Human Hepatocytes, and Can be Partially Reversed by Antiviral Therapy

Wenjing Zai[1], Kongying Hu[1], Jianyu Ye[1], Jiahui Ding[1], Chao Huang[1], Yaming Li[1], Zhong Fang[3], Min Wu[3], Cong Wang[3], Jieliang Chen[1,2], Zhenghong Yuan[1,2]

MATERIALS AND METHODS

Cells and cell culture

Cryopreserved primary human hepatocytes were purchased from BioreclamationIVT. Cells were thawed in a 37 °C water bath, then gently transferred from the vial into 3 ml pre-warmed plating medium in a 15 ml falcon. Cells were then centrifugated at 1000 rpm for 3 min. The supernatant was carefully discarded with a pipette. Cells were resuspended with additional 3 ml pre-warmed plating medium containing 10 µl DNase (5 mg/ml) and incubated at 37 °C for 10 min to remove DNA fragments from cell debris. Cells were centrifugated at 1000 rpm for 3 min, the supernatant was discarded carefully, and then gently resuspended with 3 ml 5C medium in the 15 ml falcon. Cell viability and concentration can be verified by Trypan Blue (Gibco). The cell suspensions were then transferred to a new 50 ml falcon and diluted with 5C medium into 5×10^5 cell/ml. 2.5×10^5 cells were seeded into each well of collagen I coated 24-well plates (in a volume of 500 µl). Higher or lower density of cells will not yield the ideal compact monolayer of PHHs.

Viruses

HepAD38 cells were cultured in Dulbecco's modified minimal essential medium (DMEM) supplemented with 10 % FBS. The medium was changed to DMEM consisting of 3 % FBS and 2 % DMSO when cells were confluent. Supernatants were collected every 3-4 days, pooled, and concentrated 100-fold using 7~8 % polyethylene glycol (PEG) 8000 prediction (7000 g, 30 min). The concentrated virus stock was aliquoted and stored at-80 °C.

Peptide labeling

For iTRAQ labelling, iTRAQ 8-plex reagents (AB Sciex) were dissolved in isopropanol, and each were utilized to label 100 µg peptide. Sample labelling, as indicated in Fig. S2A, was as follows: Mock-2d (iTRAQ8-113), HBV-2d (iTRAQ8-114), Mock-7d (iTRAQ8-115), HBV-7d (iTRAQ8-116), ETV-7d (iTRAQ8-117), Mock-28d (iTRAQ8-118), HBV-28d (iTRAQ8-119), ETV-28d (iTRAQ8-121). Following incubation at room temperature for 2 h, 50 µl ultrapure water were added, and stand at room temperature for another 30 min. Equal amounts of peptides were mixed and dried by vacuum for further detection.

For TMT labelling, Tandem Mass Tag 10-plex reagents (Thermo Scientific) were dissolved in anhydrous acetonitrile and 10 µl of each were added to each peptide sample

with a final acetonitrile concentration of 30% (v/v). The samples, as indicated in Fig. S2A, were labelled as follows: Mock-1 (TMT10-126), Mock-2 (TMT10-127N), Mock-3 (TMT10-127C), Mock+ETV (TMT10-128N), Mock+TDF (TMT10-128C), HBV-1 (TNT10-129N), HBV-2 (TMT10-129C), HBV-3 (TMT10-130N), HBV+ETV (TMT10-130C), HBV+TDF (TMT10-131). Samples were incubated at room temperature for 1 h, then hydroxylamine were added to a final concentration of 0.3% (v/v) to quench the reaction. Equal amounts of labelled peptides were mixed and dried for further off-line peptide fractionation.

Sample Preparation and MS Analysis

Proteomics for RNAi-treated PHHs were performed as previous described(1). For sample preparation, cells were harvested by RIPA lyses buffer supplemented with 1% protease inhibitor cocktail (v/v), then sonicated and centrifuged. The supernatants were collected and the concentration of proteins were measured by BCA analysis. Proteins were then reduced, alkylated, and digested with trypsin (enzyme/substrate ratio of 1:50) overnight. The lysates were then subjected to chemical derivatization with propionic anhydride, acidified with TFA to a final concentration of 1 % (v/v), desalted with SepPak C18 cartridges and lyophilized. The peptides were fractionated through an SCX-SPE Column, and the resulting fractions were marked as “elution 1”, “elution 2” and “elution 3”. The fractions were then lyophilized and desalted. A total of 1 μg peptides from each fraction were preserved for LC-MS/MS analysis, and the remaining peptides of “elution 1” and “elution 2” were subjected to demethylation and DMEN-amidation. The peptide (IPNFKGASHAAEQLPR) was used as the standard.

LC-MS/MS analysis were performed on a nano-HPLC chromatography system connected to a hybrid trapped ion mobility spectrometry quadrupole time of flight mass spectrometer (TIMS-TOF Pro, Bruker Daltonics, Billerica, MA) via a CaptiveSpray nano-electrospray ion source. In brief, a total of 200 ng peptides (in Solvent A (0.1 % formic acid)) was loaded and separated with a 60 min gradient (2-22 % Solvent B (ACN with 0.1 % formic acid) for 45 min, 22-37 % B for 5 min, 37-80 % B for 5 min, and 80 % B for 5 min). The flow rate was kept at 200 nL/min, the accumulation and ramp time for MS analysis were 100 ms each. Survey full-scan MS spectra (m/z 100-1700) were acquired with the ion mobility from 0.7 to 1.3 Vs/cm^2. The overall acquisition cycle of 1.16 s comprised on full TIMS-MS scan and 10 parallel accumulation-serial frag-mentation (PASEF) MS/MS scans. The collision energy was ramped linearly as a function of the mobility from 59 eV at 1/K0 = 1.6 Vs/cm^2 to 20 eV at 1/K0 = 0.6 Vs/cm^2.

The raw MS data were then searched against the Swiss-Prot database using PEAKS Online Xpro Software (v1.4) for peptide and protein identification. Mass tolerances were set as 15 ppm for parent ions and 0.05 Da for fragments. Trypsin and ArgC were selected as the protease for nonpropionylated and propionylated samples, respectively. PTM identification was performed in PEAKS PTM searching module, with a false discovery rate (FDR) at PSM and protein level controlled below 1%.

Immunoblotting

For immunoblotting, cells were lysed with RIPA buffer containing 1 × protease

inhibitor cocktail (Roche). Protein concentrations were measured by BCA (Pierce). Equal amounts of proteins were loaded and separated by SDS-PAGE, then transferred to PVDF membrane using Trans-Blot Systems (Bio-Rad). Membranes were then blocked in 5 % non-fat dried milk/0.2 % Tween and incubated with indicated primary antibodies at 4 °C overnight, washed with TBST for three times and incubated with fluorescence-labeled secondary antibodies at room temperature for 1 h. Blots were then washed for three times, and fluorescent signals were detected using LI-COR Odyssey, and images were processed using Image Studio Lite (LI-COR).

HBV cccDNA isolation

Cells were washed with PBS and lysed with 700 µl TE buffer (10 mM Tris-HCl [pH 7.5], 10 mM EDTA) and 100 µl of 10 % SDS at room temperature for 10 min. Samples were transferred to new tubes, 200 µl of 5 M NaCl were added and mixed. After incubating at 4 °C overnight, samples were centrifuged at 13 000 rpm for 30 min at 4 °C. The supernatants were then purified by phenol-chloroform (1:1) extraction and ethanol precipitation. Samples were then detected by Q-PCR using specific primers or applied to Southern Blot analysis according to the method descried previously.

Analysis of HBV integration

The incidence of HBV integration was interrogated by identifying human-HBV chimeric reads in RNA-seq dataset as previous reported (2-4). In brief, raw sequencing reads were aligned with STAR (version 2.7.1a) to human plus HBV genome sequence, the human-viral junction reads were then extracted using the read names from the STAR generated junction file by Picard (http://broadinstitute.github.io/picard). The junctions between human and HBV were visualized by Circos (http://circos.ca/). Representative chimeric reads were visualized by inspection with the UCSC BLAT tool.

Analysis of gene expression in patients

The analysis of relative gene expression levels in patients was performed as described(5). For the analysis of the correlation between gene expression with serum HBV DNA levels gene levels were assessed from GSE83148(6). Gene expression of HBV patients at different stage of virus infection was assessed from GSE65359(7). To analyze the relationships of relative gene with HBV-induced HCC, data from GSE65485 and GSE14520 were utilized(8, 9). Gene expression levels were displayed as signal intensity values.

SUPPLEMENTARY TABLES

Table S1. Interactive dataset for all proteomics and transcriptomics data, Related to Figure 1

Table S2. DAVID analysis of functional pathway enrichment, Related to Figure 2.

Table S3. K-means clustering analysis of transcriptional and post-transcriptional regulators, Related to Figure 3.

Table S4. Transcriptomics and proteomics analysis of RNAi-treated PHHs, Related to Figure 6.

Table S5. Overexpression screening of host factors that participates in viral replication or host antagonism, Related to Figure 7.

References:

1. Wang Z, Zhang L, Yuan W, Zhang Y, Lu H. 2021. SAPT, a Fast and Efficient Approach for Simultaneous Profiling of Protein N- and C-Terminome. *Analytical Chemistry* 93: 10553-60
2. Zhang L, Richards A, Barrasa MI, Hughes SH, Young RA, Jaenisch R. 2021. Reverse-transcribed SARS-CoV-2 RNA can integrate into the genome of cultured human cells and can be expressed in patient-derived tissues. *Proceedings of the National Academy of Sciences* 118: e2105968118
3. Kazachenka A, Kassiotis G. 2021. SARS-CoV-2-Host Chimeric RNA-Sequencing Reads Do Not Necessarily Arise From Virus Integration Into the Host DNA. *Frontiers in Microbiology* 12
4. Ringlander J, Skoglund C, Prakash K, Andersson ME, Larsson SB, Tang KW, Rydell GE, Abrahamsson S, Castedal M, Norder H, Hellstrand K, Lindh M. 2020. Deep sequencing of liver explant transcriptomes reveals extensive expression from integrated hepatitis B virus DNA. *Journal of Viral Hepatitis* 27: 1162-70
5. Eller C, Heydmann L, Colpitts CC, El Saghire H, Piccioni F, Jühling F, Majzoub K, Pons C, Bach C, Lucifora J, Lupberger J, Nassal M, Cowley GS, Fujiwara N, Hsieh S, Hoshida Y, Felli E, Pessaux P, Sureau C, Schuster C, Root DE, Verrier ER, Baumert TF. 2020. A genome-wide gain-of-function screen identifies CDKN2C as a HBV host factor. *Nature Communications* 11
6. Zhou W, Ma Y, Zhang J, Hu J, Zhang M, Wang Y, Li Y, Wu L, Pan Y, Zhang Y, Zhang X, Zhang X, Zhang Z, Zhang J, Li H, Lu L, Jin L, Wang J, Yuan Z, Liu J. 2017. Predictive model for inflammation grades of chronic hepatitis B: Large-scale analysis of clinical parameters and gene expressions. *Liver Int* 37: 1632-41
7. Nakagawa S, Wei L, Song WM, Higashi T, Ghoshal S, Kim RS, Bian CB, Yamada S, Sun X, Venkatesh A, Goossens N, Bain G, Lauwers GY, Koh AP, El-Abtah M, Ahmad NB, Hoshida H, Erstad DJ, Gunasekaran G, Lee Y, Yu ML, Chuang WL, Dai CY, Kobayashi M, Kumada H, Beppu T, Baba H, Mahajan M, Nair VD, Lanuti M, Villanueva A, Sangiovanni A, Iavarone M, Colombo M, Llovet JM, Subramanian A, Tager AM, Friedman SL, Baumert TF, Schwarz ME, Chung RT, Tanabe KK, Zhang B, Fuchs BC, Hoshida Y. 2016. Molecular Liver Cancer Prevention in Cirrhosis by Organ Transcriptome Analysis and Lysophosphatidic Acid Pathway Inhibition. *Cancer Cell* 30: 879-90
8. Dong H, Zhang L, Qian Z, Zhu X, Zhu G, Chen Y, Xie X, Ye Q, Zang J, Ren Z, Ji Q. 2015. Identification of HBV-MLL4 Integration and Its Molecular Basis in Chinese Hepatocellular Carcinoma. *PLoS One* 10: e123175
9. Roessler S, Jia HL, Budhu A, Forgues M, Ye QH, Lee JS, Thorgeirsson SS, Sun Z, Tang ZY, Qin LX, Wang XW. 2010. A unique metastasis gene signature enables prediction of tumor relapse in early-stage hepatocellular carcinoma patients. *Cancer Res* 70: 10202-12

Supplementary Figure 1

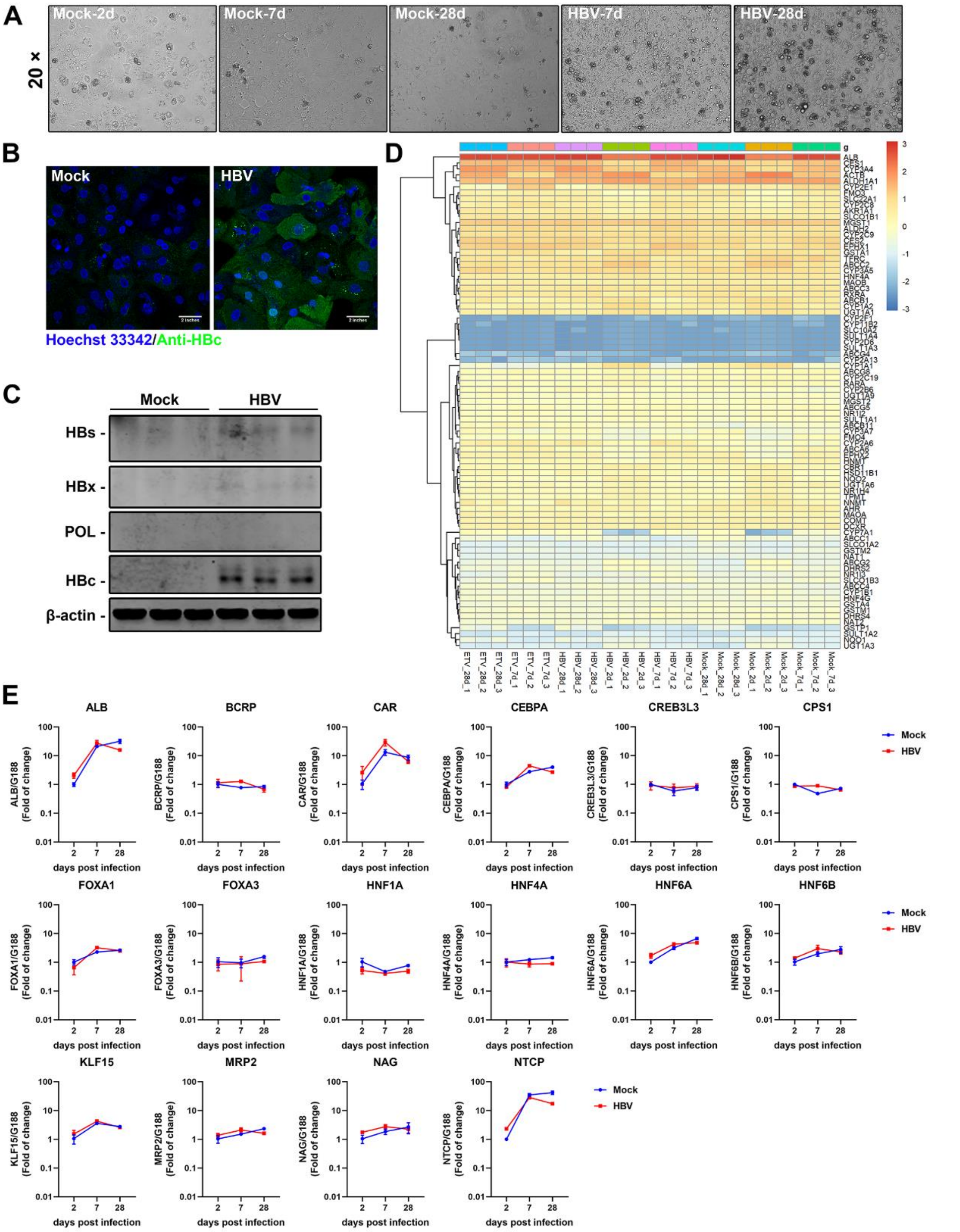

FIG S1, related to FIG 1. Hepatic characteristics of long-term cultured 5C-PHHs. (A) Representative images of cultured 5C-PHH at different time points. (B) 5C-PHH were infected with HBV at MOI of 200, and the efficiency of HBV infection was confirmed by immunofluorescence staining with anti-HBc antibody. (C) Expression levels of intracellular HBs, HBx, POL, HBc, and load-control β-actin of mock- or HBV-infected 5C-PHH cells at 28 dpi were determined by Western Blot analysis. (D) Heatmaps displaying log2-transformed gene expression profiles of drug-metabolizing, live-specific transcripts in 5C-PHH. (E) Gene expression levels of hepatic surrogate functional markers and the hepatic transcription factors of mock- or HBV- infected 5C-

PHH at indicated time points were analyzed by Q-PCR with specific primers.

Supplementary Figure 2

A

	iTRAQ	TMT
Total peptides	40585	33640
Human proteins	4916	4024
HBV proteins	1	1

Label	iTRAQ8-113	iTRAQ8-114	iTRAQ8-115	iTRAQ8-116	iTRAQ8-117	iTRAQ8-118	iTRAQ8-119	iTRAQ8-121
Sample	Mock-2d	HBV-2d	Mock-7d	HBV-7d	ETV-7d	Mock-28d	HBV-28d	ETV-28d

Label	TMT10-126	TMT10-127N	TMT10-127C	TMT10-128N	TMT10-128C	TMT10-129N	TMT10-129C	TMT10-130N	TMT10-130C	TMT10-131
Sample	Mock-1	Mock-2	Mock-3	Mock+ETV	Mock+TDF	HBV-1	HBV-2	HBV-3	HBV+ETV	HBV+TDF

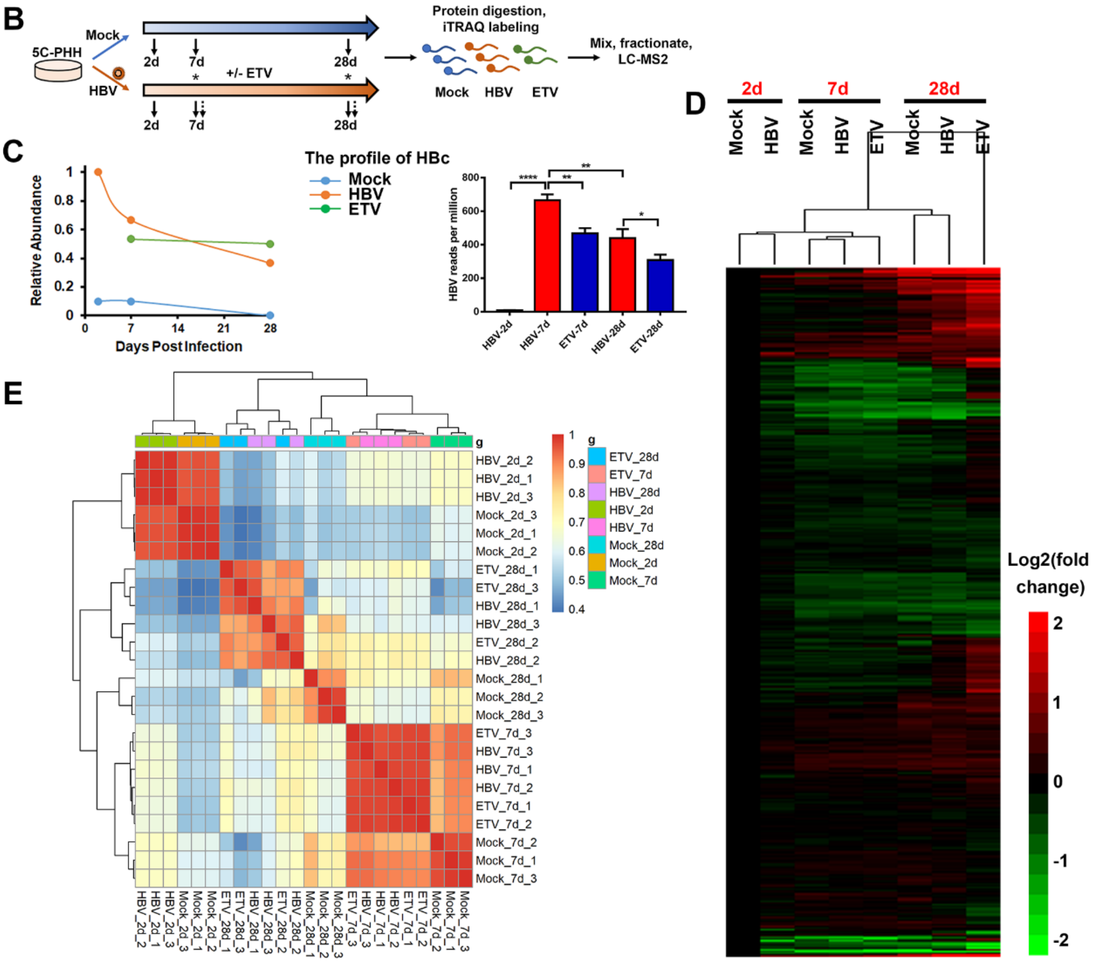

FIG S2, related to FIG 1. Basic information of proteomics and transcriptomics. (A) Numbers of human and viral proteins quantified in each experiment, and schematics of sample labelling. (B) Schematics of experiment workflow. (C) Temporal profiles of HBc in temporal proteomics. HBV reads normalized to total read counts. (D) Hierarchical cluster analysis of proteins quantified in mock- or HBV-infected cells at 2, 7 and 28 dpi, and ETV-treated HBV-infected cells at 7 and 28 dpi demonstrated that samples of the same time point clustered together. Fold change was calculated for each value compared to the mock-infected sample at 2 dpi. (E) Matrix of Person correlations for RNA-seq data.

Supplementary Figure 3

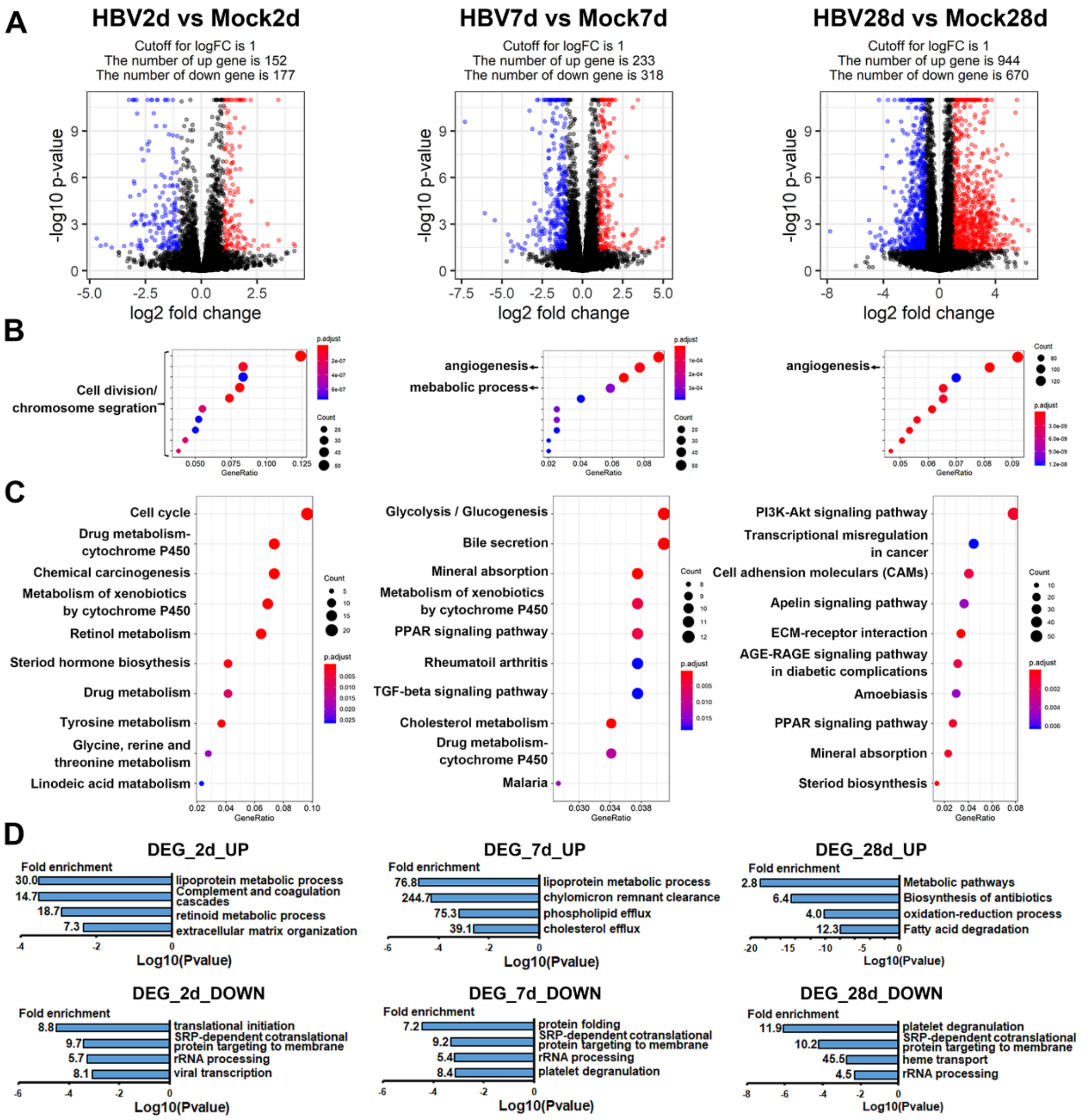

FIG S3, related to FIG 2. Temporal profiling of HBV-infected hepatocytes. (A) Volcano plot of all genes quantified in comparison between HBV-infected replicates and relative mock-infected cells. *P*-values were estimated using two-tailed *t*-test (red dots: upregulated; blue dots: downregulated; > 2-fold change and $P < 0.05$). (B) Gene Ontology "biological process" terms enriched among proteins dysregulated by HBV-infection were displayed. (C) KEGG pathway terms enriched among genes dysregulated by HBV infection at 2, 7 and 28 dpi were displayed. (D) Gene ontology "biological process" and "molecular function", and KEGG pathway terms enriched among proteins dysregulated by HBV infection at 2, 7 and 28 dpi. DAVID functional annotation clusters with *P*-values < 0.05 were shown. A background of all quantified human proteins was used. Components of each significantly enriched cluster were shown in Table S2.

Supplementary Figure 4

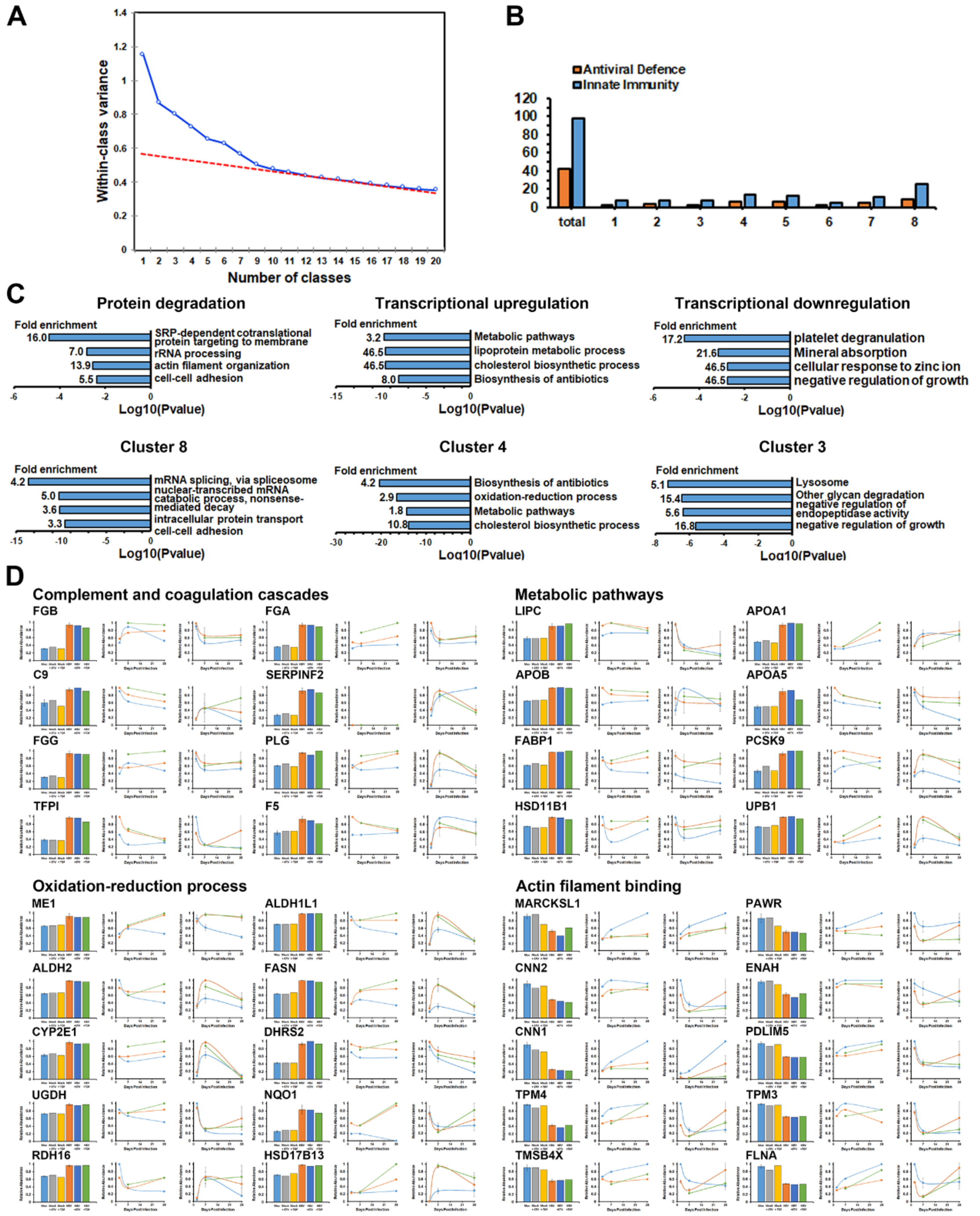

FIG S4, related to FIG 3. Transcriptional and post-transcriptional regulation of protein expression.

(A) K-means clustering with 1-20 classes was used to assess the summed distance of each protein from its cluster centroid. The point of inflexion fell between eight and nine classes, suggesting that there were at least eight distinct classes of temporal protein expression mode. (B) Numbers of proteins from the Uniprot keywords "antiviral defense" and "innate immunity" shortlists appearing in each cluster. (C) Functional annotation analysis by DAVID of proteins from "protein degradation", "transcriptional upregulation" and "transcriptional downregulation" shortlists or relative clusters. A background of all quantified proteins was used. A similar functional enrichment with

proteins in cluster 3, cluster 4 and cluster 8 was also performed. (D) Examples of proteins in "complement and coagulation cascades", "metabolic pathways", "oxidation-reduction process" and "actin filament binding" pathways in distinct screens.

Supplementary Figure 5

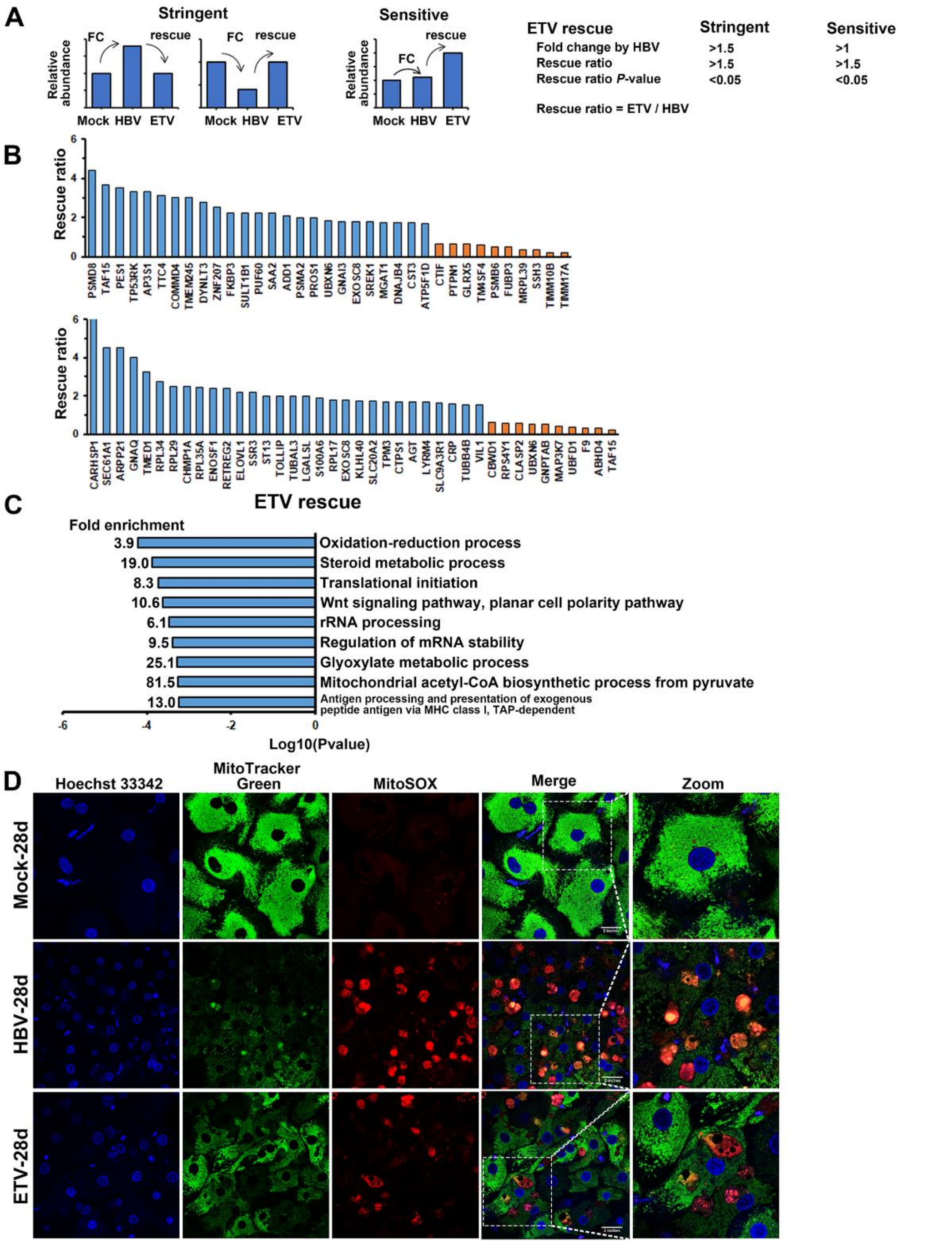

FIG S5, related to FIG 4. Identification of proteins dysregulated by HBV while recovered by nucleot(s)ide analogues.

(A) Schematic of experiment workflow. (B) Rescue ratios of all proteins identified by ETV screen at 7 dpi (upper panel) and 28 dpi (lower panel), using "stringent criteria". (C) DAVID analysis of pathway enrichment among proteins identified by ETV rescue

screen according to the "sensitive criteria". (D) Immunofluorescent analysis of Hoechst 33342 (blue), MitoTracker Green (green) and MitoSOX (red) stain in mock- and HBV-infected 5C-PHH cells with or without ETV treatment, as indicated.

Supplementary Figure 6

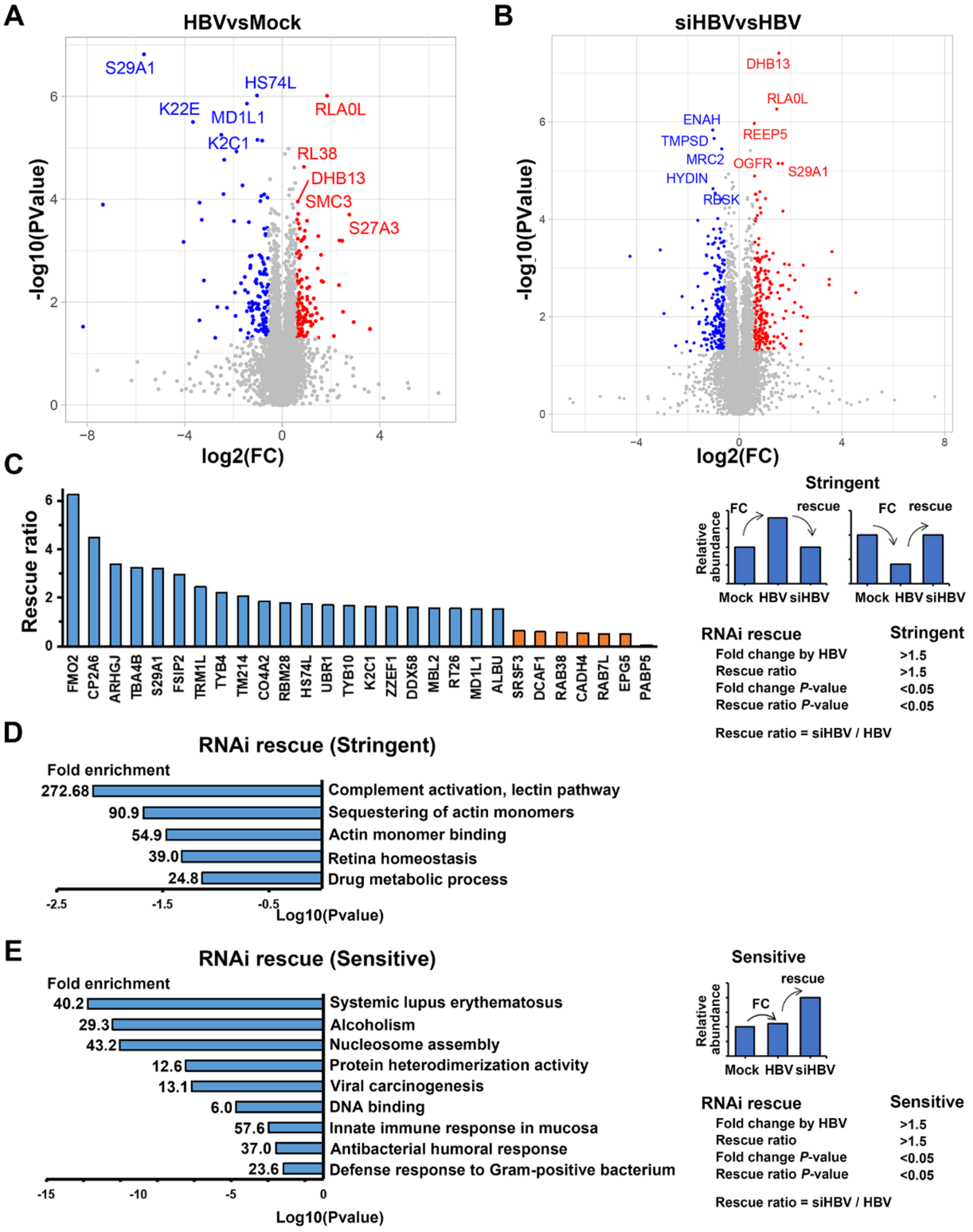

FIG S6, related to FIG 6. Blocking HBV antigen by RNAi treatment significantly relieved HBV-induced host remodeling.

(A) Volcano plot of proteins quantified in comparison between HBV-infected replicates and relative mock-infected cells, and (B) volcano plot of proteins between siHBV-treated and untreated HBV-infected cells. *P*-values were estimated using two-tailed *t*-test (red dots: upregulated; blue dots: downregulated; > 1.5-fold change and $P < 0.05$).

(C) Rescue ratios of proteins identified that could be dysregulated by HBV infection while rescued by siHBV treatment, according to the "stringent criteria" shown on the right. (D) DAVID analysis of pathway enrichment among proteins that were degraded by HBV infection while rescued by siHBV treatment. (E) DAVID analysis of pathway enrichment among proteins identified that could be rescued by siHBV treatment according to the "sensitive criteria" shown on the right.

Supplementary Figure 7

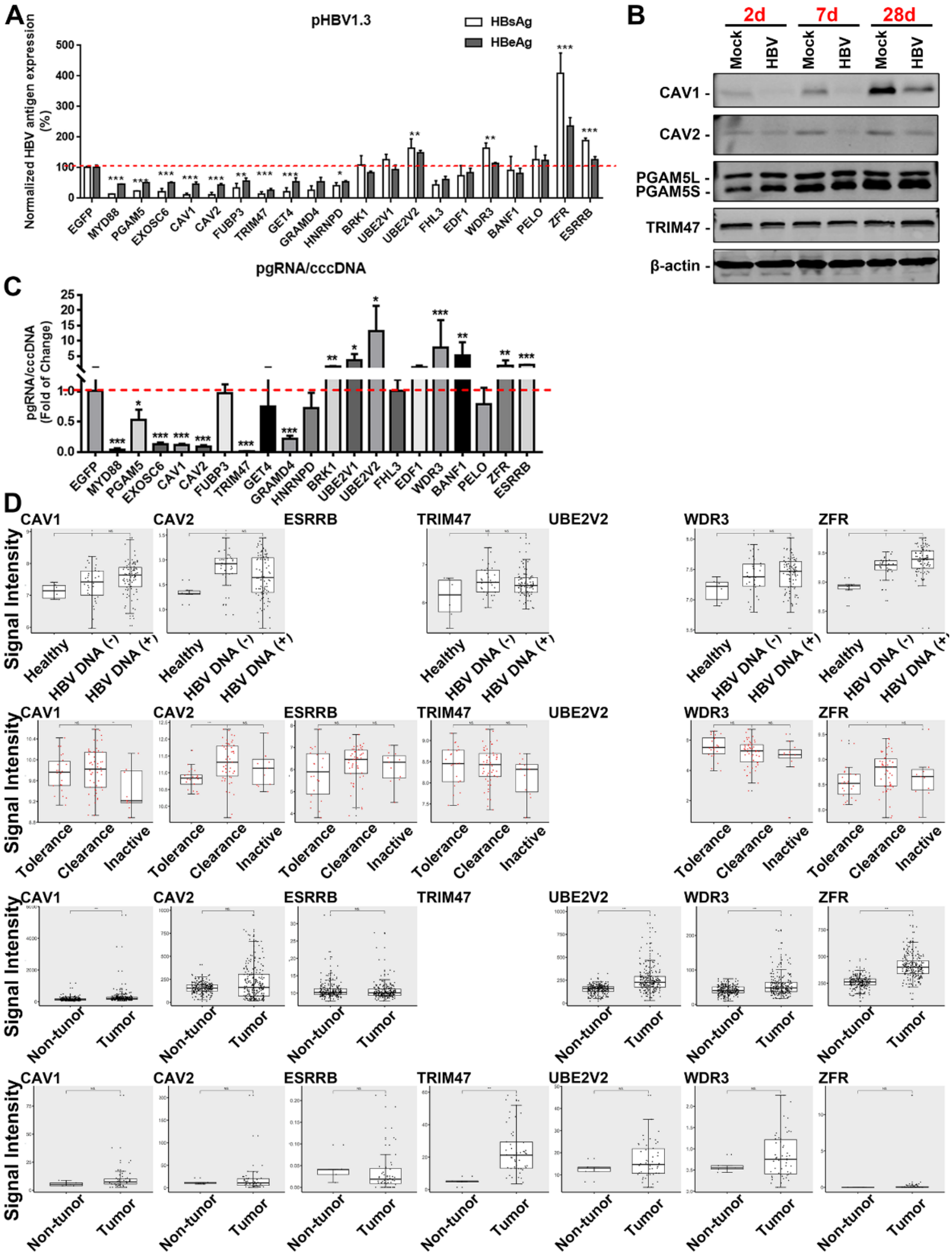

FIG S7, related to FIG 7. Overexpression screening of dysregulated proteins identified in combined analysis.

(A) Selected pro- and anti- HBV genes were co-transfected with pHBV1.3 plasmid in

HepG2 cells. HBV antigen levels were detected at 48 h post-transfection and normalized to EGFP control. (B) Representative immunoblots of CAV1, CAV2, PGAM5 and TRIM47 and load-control β-actin of mock- or HBV- infected 5C-PHH cells at indicated time points. (C) Relative HBV pgRNA/cccDNA levels were calculated and normalized to the EGFP control. (D) Gene expression levels in HBV-infected patients with undetectable or detectable HBV DNA comparing to healthy patients (HBV DNA (-), n = 32, HBV DNA (+), n = 90); gene expression levels in HBV-infected patients at different stage of disease (tolerance, n = 22; clearance, n = 50; inactive, n = 11); and gene expression in tumors and adjacent tissues in HBV-associated HCC patients from two different cohorts (cohort1: non-tumor, n =198, tumor, n = 98; cohort2: non-tumor, n =5, tumor, n = 50). For more details, see “materials and methods”.

Supplementary Figure 8

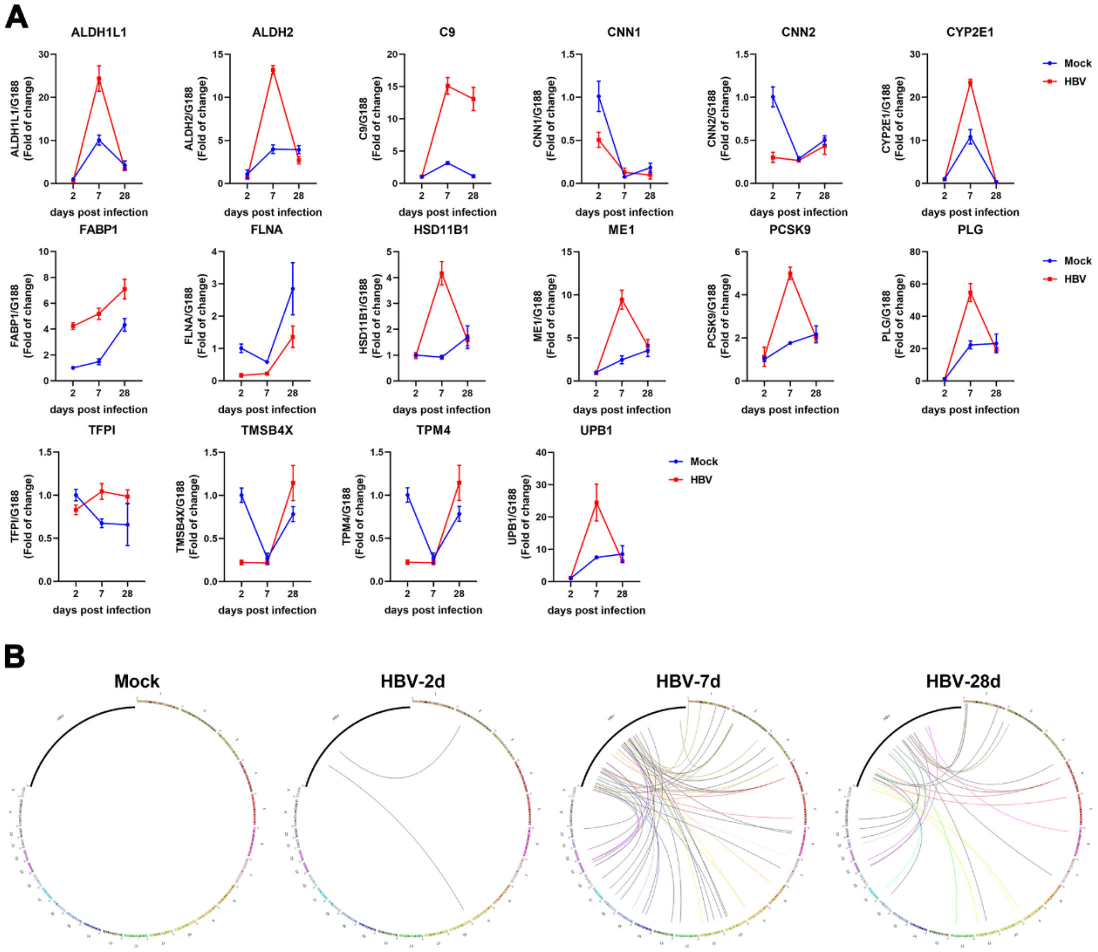

C

E00552:372:H3F2NCCX2:1:1120:16549:24286

AAAAATGTCATGTTCCTCAAGTAGAGCAGCAAAGTTCTTGCTAATGAGTGTCCCCAACCTCCAATCACTCACCAACCTCCTGTC
CTCCAACTTGTCCTGGTTATCGCTGGATGTGTCTGCGGCGTTTTATCATCTTCCTCTTCATCCTGC

E00552:372:H3F2NCCX2:1:1111:23145:4386

ATTAAAGGTCTTTGTACTAGGAGGCTGTAGGCATAAATTGGTCTGCGCACCAGCACCATGCAACTTTTTCACCTCTGTCTTTCCC
CATGGCCTTGCAAGCCAGGGTGGCTTTGCAGCTTGCTACTCACGTAAGC

E00491:444:H3C7GCCX2:2:1204:11403:37313

CAGCCGATGTCTGGAGCCTGGGCGTGGCGCTCTTCACCATGCTGGCCGGTAAAATTCAGAGAGTAACCCCATCTCTTTGTTTTG
TTAGGGTTTAAATGTATACCCAAAGACAAAAGAAAATTGGTAAAAGCGGTAAAAAGGGACTCAAG

E00552:372:H3F2NCCX2:1:1120:16549:24286

AGCACGGGACCATGCCGAACCTGCATGACTACTGCTCAAGGAACCTCTATGTATCCCTCCTGTTGCTGTACCAAACCTTCGGAC
GGAAATTGCACCTGTATTCCCATCTCGCTTCTCCCGGTGTGGTGCTCCGCTGTTATTCCTGCCATC

FIG S8, related to FIG 8. Cytopathic effects and HBV integration caused by long-term HBV infection.

(A) Expression levels of genes of mock- or HBV- infected 5C-PHH cells associated with **Figure S4D** were analyzed by Q-PCR with specific primers. (B) Human-HBV chimeric junctions were analyzed and visualized by Circos. (C) Representative examples of human-HBV chimeric reads. HBV- and human- derived reads were shown in red and blue, respectively. Overlapping sequence were shown in green.
